# Resolution of constipation, anal stricture, and iron deficiency anemia after iron infusion: an analogy with Plummer Vinson syndrome

**DOI:** 10.1186/s40064-016-3629-8

**Published:** 2016-11-15

**Authors:** Vijaypal Arya, Shikha Singh, Shashank Agarwal, Arjun Ohri

**Affiliations:** 1 Hofstra Northwell School of Medicine, Hofstra University, Hempstead, NY USA; 2 Division of Gastroenterology, Wyckoff Heights Medical Center, Brooklyn, NY USA; 3 NYU Langone Medical Center, Manhattan, NY USA; 4 Vijaypal Arya Physician P.C., 7554 Metropolitan Avenue, New York, NY USA

**Keywords:** Plummer Vinson syndrome, Iron deficiency anemia, Anal stricture

## Abstract

**Background:**

Anal stricture is a disabling condition which is often unresponsive to conservative medical management. The complications of surgical procedures such as dilatations and anoplasty make it a formidable treatment challenge. Through this case, we report and explore a new medical treatment for ano-rectal strictures with an analogy to Plummer Vinson syndrome.

A 69-year-old male presented with chronic constipation, rectal pain, and easy fatigability. The physical exam was negative for anal fissure and a digital rectal examination could not be completed because an index finger could not be advanced through the narrowed anus. Laboratory reports revealed microcytic hypochromic anemia with iron deficiency. A colonoscopy performed with a GIF XQ180 OLYMPUS scope, confirmed anal stricture with non-specific colitis. Conservative management with laxatives, high fiber diet, local anesthetics with a trial of mesalamine was initiated but the patient continued to have symptoms. He was referred to a hematologist for an evaluation of anemia and was started on intravenous (IV) iron infusion.

**Findings:**

The patient’s symptoms of constipation, anal stricture and iron deficiency anemia resolved with iron infusion over 3 months. A repeat rectal exam was painless and confirmed resolution of anal stricture.

**Conclusion:**

IV iron supplementation combined with conventional anal dilatation presents as a promising approach toward the treatment of anal strictures.

## Case report

A 69-year-old male presented with chronic constipation, rectal pain, and easy fatigability. The patient had no history of hemorrhoids, inflammatory bowel disease, or thyroid disorder and did not report any rectal bleeding. His treatment history was negative for laxative abuse and opiates. His dietary assessment revealed a diet that was rich in fiber and vegetables. The physical exam revealed conjunctival pallor, and laboratory results were positive for microcytic hypochromic anemia (Table 1). The patient’s anal canal was carefully examined after gentle traction of the glutei. It was negative for certain conditions, such as anal fissure or a thrombosed hemorrhoid, which frequently present with rectal pain. A digital rectal examination (DRE) could not be completed because an index finger could not be advanced through the narrowed anus. An anoscopy was not performed. Instead, the patient was scheduled for a colonoscopy for further examination and evaluation of anemia.

**Table 1:** Lab values pre and post iron replacement therapy

	Pre-iron replacement therapy	Post-iron replacement therapy
Hb (g/dL)	9.4	12.1
Ht (%)	31.6	38.3
MCV (fl)	71.8	84
MCH (pg)	21.4	26.5
MCHC (g/dL)	29.9	31.8
RDW (%)	19.1	13.2
Platelets (Thous/mcL)	320	218
WBC (Thous/mcL)	4.2	6.1
ALT (U/L)	10	5
AST (U/L)	30	21

**Fig. 1:**
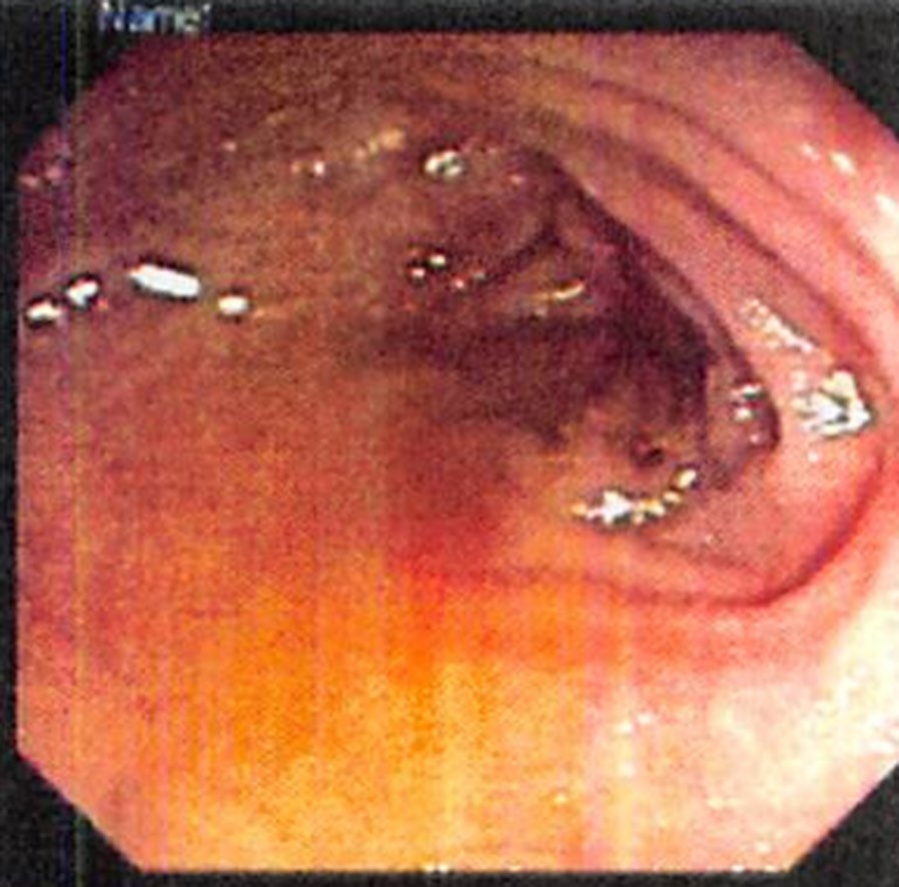
The non-specific inflammation noted at the recto-sigmoid junction

A complete work-up of anemia was performed to ascertain the cause of the patient’s iron deficiency. An upper gastrointestinal endoscopy with a GIF-Q 180 OLYMPUS scope was negative for upper gastrointestinal bleeding. A markedly narrowed anal canal prevented insertion of a standard colonoscope. Therefore, the procedure was performed with a GIF-Q 180 OLYMPUS scope, which confirmed anal stricture with non-specific colitis at the recto-sigmoid junction (Fig. 1). A biopsy of colonic mucosa did not reveal active inflammation or distortion of cryptal architecture. A CT scan of the abdomen was unremarkable. A capsule endoscopy did not reveal any small bowel source of GI bleeding. His celiac panel was normal, and there was no evidence suggestive of any other cause of anemia. Conservative management with laxatives, a high fiber diet, and local anesthetics were initiated along with a trial of mesalamine for unspecific inflammation. However, the patient continued to have symptoms of constipation and rectal pain. A trial of nitroglycerine 0.4% also failed to resolve the patient’s symptoms. The patient was referred to a hematologist for further evaluation of anemia and was started on IV iron infusions. After three months of treatment, the patient’s anemia and complaints of constipation and rectal pain had resolved dramatically (Table 1). Resolution of anal stenosis was confirmed with a “painless” DRE and repeat colonoscopy, which revealed absence of an anal stricture.

## Discussion

Anal stricture is an uncommon and disabling condition. Ninety percent of anal strictures are caused by overzealous hemorroidectomies (Brisinda 2000). Treatment of anal strictures can be unresponsive to conservative management, and the complications of dilatations and anoplasty make it a formidable treatment challenge.

The triad of constipation, anal stricture, and IDA in this case report describe an unknown parallel to PVS, which has the classic presentation of dysphagia, esophageal webs, and IDA. The patient’s symptoms of constipation and anal stricture not responding to conservative treatment resolved with IV iron treatment, which has long been known to resolve dysphagia and esophageal webs in PVS (Tahara et al. 2014). Conservative management is recommended for mild to moderate stenosis; conversely, severe stenosis should be treated with anoplasty (except for cases of Crohn’s colitis, where anoplasty is an absolute contraindication) (Liberman and Thorson 2000).

Rectal pain that stemmed from an ano-rectal fissure was an important differential in this scenario; however, the patient’s negative history for any signs of rectal bleeding and negative physical exam made it less probable. Notably, the formation of an anal stricture, which was confirmed with a DRE and colonoscopy in this patient with chronic constipation, may also be explained by fibrosis secondary to a spontaneously healed anal fissure. Multiple mechanisms have been reported regarding the role of iron deficiency in causing dysphagia and esophageal webs. Shamma’a et al. showed a 66 % association between iron deficiency and esophageal stenosis (Shamma’a and Benedict 1958). Along with nonsteroidal anti-inflammatory drugs (NSAIDS), IDA has also played a role in the formation of diaphragm-like strictures in the small intestine, duodenum, and stomach (Wang 2011). A possible histophysiological mechanism is vascular ischemia tissue hypoxia, leading to mucosal injury and the formation of strictures post-fibrotic repair. We propose that the formation of ano-rectal strictures in this patient with IDA can also be explained through a similar mechanism—the component of the same alimentary tract, both anatomically and physiologically. Previous studies have implied a possible relationship between high cell turnover in the alimentary tract and tissue iron deficiency (Jacobs 1969). Hence, iron deficiency may be imperative in producing motility dysfunction, leading to dysphagia in the foregut and constipation in our patient.

In conclusion, this analogy with PVS suggests that there are treatment benefits from iron infusion in refractory cases of anal stenosis, constipation, and anemia. IV iron supplementation combined with conventional anal dilatation presents as a promising approach toward the treatment of anal strictures. This report further presents another intellectual challenge for our medical fraternity.

## Abbreviations

PVS: Plummer Vinson syndrome
IDA: iron deficiency anemia
DRE: digital rectal examination
IV: intravenous
NSAIDS: nonsteroidal anti-inflammatory drugs
